# 16-[(*E*)-Benzyl­idene]-13-hy­droxy-4-methyl-2-phenyl-4,14-diaza­penta­cyclo-[12.3.1.0^1,5^.0^5,13^.0^7,12^]octa­deca-7(12),8,10-triene-6,17-dione

**DOI:** 10.1107/S1600536810020271

**Published:** 2010-06-05

**Authors:** Raju Suresh Kumar, Hasnah Osman, Mohamed Ashraf Ali, Ching Kheng Quah, Hoong-Kun Fun

**Affiliations:** a School of Chemical Sciences, Universiti Sains Malaysia, 11800 USM, Penang, Malaysia; b Institute for Research in Molecular Medicine, Universiti Sains Malaysia, 11800 USM, Penang, Malaysia; c X-ray Crystallography Unit, School of Physics, Universiti Sains Malaysia, 11800 USM, Penang, Malaysia

## Abstract

In the title compound, C_30_H_26_N_2_O_3_, the two pyrrolidine rings adopt twisted and envelope conformations, whereas the cyclo­pentane ring adopts an envelope conformation. The least-squares planes through the pyrrolidine rings form a dihedral angle of 41.72 (10)°. The mol­ecular structure is stabilized by an intra­molecular O—H⋯N hydrogen bond, which generates an *S*(5) ring motif. Centrosymmetrically related mol­ecules are linked *via* two pairs of inter­molecular C—H⋯O inter­actions, forming *R*_2_^2^(16) ring motifs. In the crystal packing, the mol­ecules are linked into two-dimensional networks parallel to the *ab* plane *via* C—H⋯O inter­actions.

## Related literature

For general background to and the biological activity of pyrrolidine derivatives, see: Gothelf & Jørgensen (1998 ▶); Gu *et al.* (2004 ▶); Horri *et al.* (1986 ▶); Tsukamoto *et al.* (1989 ▶); Karpas *et al.* (1988 ▶). For the biological activity of heterocycles with piperidine sub-structures, see: El-Subbagh *et al.* (2000 ▶); Dimmock *et al.* (2001 ▶); Lee *et al.* (2001 ▶). For reference bond lengths, see: Allen *et al.* (1987 ▶). For the stability of the temperature controller used for the data collection, see: Cosier & Glazer (1986 ▶). For hydrogen-bond motifs, see: Bernstein *et al.* (1995 ▶). For ring conformations, see: Cremer & Pople (1975 ▶).
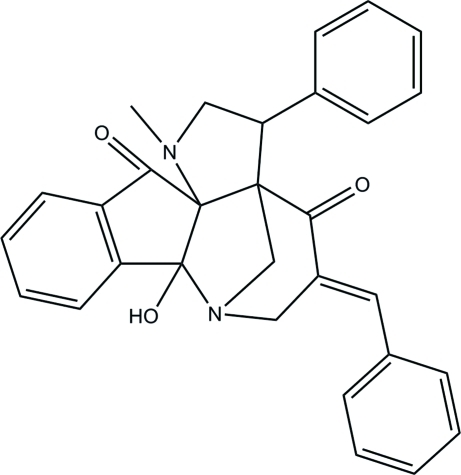


## Experimental

### 

#### Crystal data

C_30_H_26_N_2_O_3_
*M* *_r_* = 462.53
Triclinic, 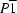

*a* = 9.0333 (5) Å
*b* = 9.4222 (5) Å
*c* = 14.0290 (7) Å
α = 80.943 (2)°
β = 78.034 (1)°
γ = 80.578 (1)°
*V* = 1142.88 (10) Å^3^
*Z* = 2
Mo *K*α radiation
μ = 0.09 mm^−1^
*T* = 100 K
0.51 × 0.39 × 0.10 mm

#### Data collection

Bruker APEXII DUO CCD area-detector diffractometer
Absorption correction: multi-scan (*SADABS*; Bruker, 2009 ▶) *T*_min_ = 0.957, *T*_max_ = 0.991
21768 measured reflections
4213 independent reflections
3812 reflections with *I* > 2σ(*I*)
*R*_int_ = 0.026

#### Refinement

*R*[*F* ^2^ > 2σ(*F* ^2^)] = 0.043
*wR*(*F* ^2^) = 0.130
*S* = 1.06
4213 reflections
420 parameters
All H-atom parameters refined
Δρ_max_ = 0.58 e Å^−3^
Δρ_min_ = −0.29 e Å^−3^

### 

Data collection: *APEX2* (Bruker, 2009 ▶); cell refinement: *SAINT* (Bruker, 2009 ▶); data reduction: *SAINT*; program(s) used to solve structure: *SHELXTL* (Sheldrick, 2008 ▶); program(s) used to refine structure: *SHELXTL*; molecular graphics: *SHELXTL*; software used to prepare material for publication: *SHELXTL* and *PLATON* (Spek, 2009 ▶).

## Supplementary Material

Crystal structure: contains datablocks global, I. DOI: 10.1107/S1600536810020271/rz2455sup1.cif

Structure factors: contains datablocks I. DOI: 10.1107/S1600536810020271/rz2455Isup2.hkl

Additional supplementary materials:  crystallographic information; 3D view; checkCIF report

## Figures and Tables

**Table 1:** Hydrogen-bond geometry (Å, °)

*D*—H⋯*A*	*D*—H	H⋯*A*	*D*⋯*A*	*D*—H⋯*A*
O2—H12*O*⋯N2	0.82 (3)	2.10 (3)	2.6741 (19)	127 (2)
C17—H17*A*⋯O1^i^	0.965 (19)	2.56 (2)	3.278 (2)	130.8 (15)
C26—H26*A*⋯O1^ii^	0.98 (3)	2.60 (2)	3.535 (2)	161.6 (18)
C29—H29*A*⋯O2^iii^	0.96 (3)	2.43 (2)	3.363 (2)	165.7 (18)

Symmetry codes: (i) 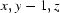; (ii) 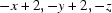; (iii) 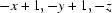.

**Fig. 1.**
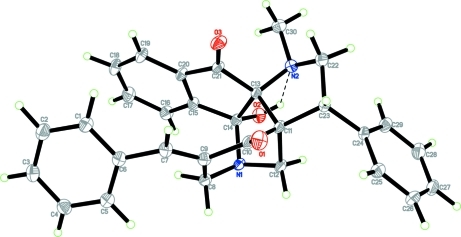
The molecular structure of the title compound, showing 50% probability displacement ellipsoids for non-H atoms and the atom-numbering scheme. Intramolecular hydrogen interaction is shown as dashed line.

**Fig. 2.**
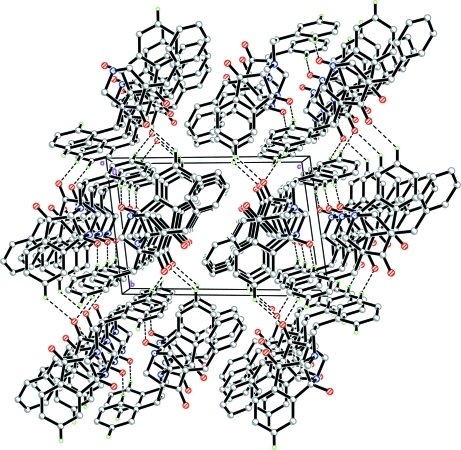
The crystal structure of the title compound viewed along the a axis. H atoms not involved in intermolecular interactions (dashed lines) have been omitted for clarity.

## supplementary crystallographic information

### Comment

The cycloaddition reaction of azomethine ylide 1,3-dipoles with olefinic dipolarophiles constitutes a straightforward approach to the synthesis of highly substituted pyrrolidine derivatives (Gothelf & Jørgensen, 1998). Pyrrolidine ring is present in many biologically active natural compounds and pharmaceuticals (Gu *et al.*, 2004), and find utility in the treatment of diseases such as diabetes (Horri *et al.*, 1986), cancer (Tsukamoto *et al.*, 1989) and viral infections (Karpas *et al.*, 1988). Heterocycles with piperidine sub-structures display important biological activities, such as cytotoxic (El-Subbagh *et al.*, 2000) and anticancer (Dimmock *et al.*, 2001) besides being useful as synthons in the construction of alkaloid natural products (Lee *et al.*, 2001).

The bond lengths (Allen *et al.*, 1987) and angles in the title compound (Fig. 1) are within normal ranges. For the two pyrrolidine rings, N1/C11–C14 is twisted about the N1–C12 with the puckering parameters (Cremer & Pople, 1975) Q = 0.4589 (16) Å and φ = 202.6 (2)° whereas the N2/C11/C13/C22/C23 ring adopts an envelope conformation with atom N2 deviating by 0.251 (1) Å from the mean plane through the remaining atoms (puckering parameters Q = 0.3814 (18) Å and φ = 356.2 (3)°). The cyclopentane (C13–C15/C20/C21) ring adopts an envelope conformation with the flap at atom C13 (puckering parameters Q = 0.2688 (10) Å and φ = 186.5 (10)°). The two pyrrolidine rings make a dihedral angle of 41.72 (10)° between their least-squares planes. The molecular structure is stabilized by intramolecular O2—H12O···N2 hydrogen bond which generates an *S*(5) ring motif (Bernstein *et al.*, 1995).

Centrosymmetrically related molecules are linked *via* two pairs of intermolecular C26—H26A···O1 and C29—H29A···O2 interactions, forming *R*_2_^2^ (16) ring motifs (Table 1). In the crystal packing (Fig. 2), the molecules are linked into two- dimensional networks parallel to the *ab* plane *via* C17—H17A···O1 interactions.

### Experimental

A mixture of 3,5-bis[(*E*)-benzylidene]tetrahydro-4(1*H*)-pyridinone (0.100 g, 0.364 mmol), ninhydrin (0.065 g, 0.364 mmol) and sarcosine (0.032 g, 0.364 mmol) were dissolved in methanol (10 ml) and refluxed for 1 h. After completion of the reaction as evident from TLC, the mixture was poured into water (50 ml). The precipitated solid was filtered and washed with water to obtain the product which was recrystallised from ethyl acetate to give the title compound as yellow crystals.

### Refinement

All H atoms were located in a difference Fourier map and refined freely. The highest residual electron density peak is located at 1.10 Å from H22A and the deepest hole is located at 0.65 Å from C22.

### Crystal data

**Table:**

C_30_H_26_N_2_O_3_	*Z* = 2
*M**_r_* = 462.53	*F*(000) = 488
Triclinic, *P*1	*D*_x_ = 1.344 Mg m^−^^3^
Hall symbol: -P 1	Mo *K*α radiation, λ = 0.71073 Å
*a* = 9.0333 (5) Å	Cell parameters from 9987 reflections
*b* = 9.4222 (5) Å	θ = 2.8–35.8°
*c* = 14.0290 (7) Å	µ = 0.09 mm^−^^1^
α = 80.943 (2)°	*T* = 100 K
β = 78.034 (1)°	Plate, yellow
γ = 80.578 (1)°	0.51 × 0.39 × 0.10 mm
*V* = 1142.88 (10) Å^3^	

### Data collection

**Table:**

Bruker APEXII DUO CCD area-detector diffractometer	4213 independent reflections
Radiation source: fine-focus sealed tube	3812 reflections with *I* > 2σ(*I*)
graphite	*R*_int_ = 0.026
φ and ω scans	θ_max_ = 25.5°, θ_min_ = 1.5°
Absorption correction: multi-scan (*SADABS*; Bruker, 2009)	*h* = −10→10
*T*_min_ = 0.957, *T*_max_ = 0.991	*k* = −11→11
21768 measured reflections	*l* = −16→16

### Refinement

**Table:**

Refinement on *F*^2^	Primary atom site location: structure-invariant direct methods
Least-squares matrix: full	Secondary atom site location: difference Fourier map
*R*[*F*^2^ > 2σ(*F*^2^)] = 0.043	Hydrogen site location: inferred from neighbouring sites
*wR*(*F*^2^) = 0.130	All H-atom parameters refined
*S* = 1.06	*w* = 1/[σ^2^(*F*_o_^2^) + (0.0728*P*)^2^ + 0.7251*P*] where *P* = (*F*_o_^2^ + 2*F*_c_^2^)/3
4213 reflections	(Δ/σ)_max_ = 0.001
420 parameters	Δρ_max_ = 0.58 e Å^−^^3^
0 restraints	Δρ_min_ = −0.29 e Å^−^^3^

### Special details

**Table:**

Experimental. The crystal was placed in the cold stream of an Oxford Cyrosystems Cobra open-flow nitrogen cryostat (Cosier & Glazer, 1986) operating at 100.0 (1) K.
Geometry. All esds (except the esd in the dihedral angle between two l.s. planes) are estimated using the full covariance matrix. The cell esds are taken into account individually in the estimation of esds in distances, angles and torsion angles; correlations between esds in cell parameters are only used when they are defined by crystal symmetry. An approximate (isotropic) treatment of cell esds is used for estimating esds involving l.s. planes.
Refinement. Refinement of *F*^2^ against ALL reflections. The weighted *R*-factor wR and goodness of fit *S* are based on *F*^2^, conventional *R*-factors *R* are based on *F*, with *F* set to zero for negative *F*^2^. The threshold expression of *F*^2^ > σ(*F*^2^) is used only for calculating *R*-factors(gt) etc. and is not relevant to the choice of reflections for refinement. *R*-factors based on *F*^2^ are statistically about twice as large as those based on *F*, and *R*- factors based on ALL data will be even larger.

### Fractional atomic coordinates and isotropic or equivalent isotropic displacement parameters (Å^2^)

**Table:**

	*x*	*y*	*z*	*U*_iso_*/*U*_eq_	
O1	0.82023 (14)	0.80718 (12)	0.24270 (9)	0.0260 (3)	
O2	0.64023 (14)	0.37335 (13)	0.07491 (8)	0.0233 (3)	
O3	0.49620 (13)	0.63815 (12)	0.34310 (8)	0.0233 (3)	
N1	0.85499 (15)	0.43423 (14)	0.12050 (9)	0.0178 (3)	
N2	0.48231 (16)	0.62423 (16)	0.12640 (10)	0.0245 (3)	
C1	0.8502 (2)	0.36212 (19)	0.51299 (12)	0.0239 (4)	
C2	0.8939 (2)	0.23665 (19)	0.57195 (13)	0.0277 (4)	
C3	1.0315 (2)	0.15119 (19)	0.54275 (13)	0.0260 (4)	
C4	1.1253 (2)	0.19167 (19)	0.45416 (13)	0.0253 (4)	
C5	1.0797 (2)	0.31676 (19)	0.39408 (12)	0.0242 (4)	
C6	0.94117 (19)	0.40277 (17)	0.42240 (12)	0.0208 (3)	
C7	0.88836 (18)	0.53511 (17)	0.36091 (12)	0.0205 (3)	
C8	0.94062 (18)	0.42773 (17)	0.19982 (11)	0.0185 (3)	
C9	0.88818 (17)	0.55001 (16)	0.26407 (12)	0.0184 (3)	
C10	0.81293 (18)	0.69051 (16)	0.21854 (11)	0.0186 (3)	
C11	0.72678 (18)	0.67304 (16)	0.13952 (11)	0.0184 (3)	
C12	0.84420 (18)	0.58165 (16)	0.06730 (11)	0.0190 (3)	
C13	0.60692 (17)	0.56650 (16)	0.18033 (11)	0.0171 (3)	
C14	0.69153 (18)	0.41489 (16)	0.15331 (11)	0.0181 (3)	
C15	0.65722 (18)	0.31123 (17)	0.24635 (11)	0.0187 (3)	
C16	0.69109 (19)	0.16143 (17)	0.25931 (13)	0.0229 (4)	
C17	0.6500 (2)	0.08829 (18)	0.35298 (13)	0.0260 (4)	
C18	0.5782 (2)	0.16322 (19)	0.43228 (13)	0.0263 (4)	
C19	0.54172 (19)	0.31276 (18)	0.41913 (12)	0.0229 (4)	
C20	0.58088 (18)	0.38532 (16)	0.32502 (11)	0.0182 (3)	
C21	0.55375 (17)	0.54298 (17)	0.29254 (11)	0.0174 (3)	
C22	0.4708 (2)	0.78239 (19)	0.11858 (14)	0.0287 (4)	
C23	0.63604 (19)	0.81395 (18)	0.09578 (12)	0.0215 (3)	
C24	0.70024 (18)	0.86012 (16)	−0.01149 (12)	0.0198 (3)	
C25	0.8288 (2)	0.93160 (19)	−0.03534 (13)	0.0259 (4)	
C26	0.8949 (2)	0.97029 (19)	−0.13279 (14)	0.0283 (4)	
C27	0.8334 (2)	0.93817 (18)	−0.20774 (13)	0.0275 (4)	
C28	0.7038 (2)	0.86923 (19)	−0.18502 (13)	0.0271 (4)	
C29	0.6380 (2)	0.83022 (17)	−0.08779 (12)	0.0231 (4)	
C30	0.3337 (2)	0.5723 (2)	0.16840 (14)	0.0283 (4)	
H1A	0.751 (2)	0.424 (2)	0.5344 (14)	0.027 (5)*	
H2A	0.824 (3)	0.207 (2)	0.6373 (17)	0.035 (6)*	
H3A	1.062 (3)	0.062 (2)	0.5834 (16)	0.035 (6)*	
H4A	1.217 (3)	0.135 (2)	0.4338 (15)	0.027 (5)*	
H5A	1.147 (2)	0.344 (2)	0.3286 (16)	0.030 (5)*	
H7A	0.839 (2)	0.624 (2)	0.3991 (15)	0.031 (5)*	
H8A	0.936 (2)	0.335 (2)	0.2384 (14)	0.023 (5)*	
H8B	1.052 (2)	0.431 (2)	0.1686 (14)	0.022 (5)*	
H12A	0.945 (2)	0.616 (2)	0.0488 (13)	0.019 (4)*	
H12B	0.802 (2)	0.5839 (19)	0.0082 (14)	0.017 (4)*	
H16A	0.742 (2)	0.114 (2)	0.2045 (14)	0.019 (4)*	
H17A	0.674 (2)	−0.016 (2)	0.3623 (15)	0.031 (5)*	
H18A	0.557 (2)	0.110 (2)	0.4966 (16)	0.031 (5)*	
H19A	0.489 (2)	0.366 (2)	0.4725 (15)	0.024 (5)*	
H22A	0.406 (3)	0.812 (3)	0.1827 (18)	0.045 (6)*	
H22B	0.407 (2)	0.828 (2)	0.0667 (15)	0.027 (5)*	
H23A	0.643 (2)	0.891 (2)	0.1339 (15)	0.028 (5)*	
H25A	0.862 (2)	0.959 (2)	0.0191 (16)	0.032 (5)*	
H26A	0.983 (3)	1.023 (2)	−0.1492 (15)	0.033 (5)*	
H27A	0.879 (3)	0.966 (2)	−0.2779 (17)	0.035 (6)*	
H28A	0.653 (3)	0.851 (2)	−0.2365 (17)	0.041 (6)*	
H29A	0.549 (3)	0.782 (2)	−0.0749 (16)	0.035 (5)*	
H30A	0.260 (2)	0.616 (2)	0.1267 (16)	0.031 (5)*	
H30B	0.284 (2)	0.607 (2)	0.2355 (16)	0.029 (5)*	
H30C	0.347 (2)	0.457 (2)	0.1750 (15)	0.031 (5)*	
H12O	0.589 (3)	0.446 (3)	0.0529 (18)	0.043 (7)*	

### Atomic displacement parameters (Å^2^)

**Table:**

	*U*^11^	*U*^22^	*U*^33^	*U*^12^	*U*^13^	*U*^23^
O1	0.0343 (7)	0.0153 (6)	0.0296 (6)	−0.0057 (5)	−0.0063 (5)	−0.0034 (5)
O2	0.0284 (6)	0.0247 (6)	0.0191 (6)	−0.0035 (5)	−0.0075 (5)	−0.0057 (5)
O3	0.0265 (6)	0.0194 (6)	0.0213 (6)	−0.0018 (5)	0.0029 (5)	−0.0055 (5)
N1	0.0205 (7)	0.0159 (6)	0.0154 (6)	−0.0033 (5)	0.0004 (5)	−0.0011 (5)
N2	0.0205 (7)	0.0265 (7)	0.0235 (7)	0.0010 (6)	−0.0038 (6)	0.0006 (6)
C1	0.0257 (9)	0.0246 (8)	0.0222 (8)	−0.0046 (7)	−0.0046 (7)	−0.0039 (7)
C2	0.0320 (9)	0.0276 (9)	0.0235 (9)	−0.0087 (7)	−0.0043 (7)	0.0005 (7)
C3	0.0333 (10)	0.0214 (8)	0.0250 (9)	−0.0047 (7)	−0.0111 (7)	0.0004 (7)
C4	0.0253 (9)	0.0241 (8)	0.0270 (9)	−0.0007 (7)	−0.0070 (7)	−0.0052 (7)
C5	0.0256 (9)	0.0252 (8)	0.0224 (8)	−0.0059 (7)	−0.0038 (7)	−0.0033 (7)
C6	0.0253 (8)	0.0195 (8)	0.0204 (8)	−0.0073 (6)	−0.0061 (6)	−0.0038 (6)
C7	0.0221 (8)	0.0176 (8)	0.0233 (8)	−0.0057 (6)	−0.0049 (6)	−0.0033 (6)
C8	0.0192 (8)	0.0153 (7)	0.0196 (8)	−0.0025 (6)	−0.0009 (6)	−0.0015 (6)
C9	0.0179 (7)	0.0153 (7)	0.0226 (8)	−0.0055 (6)	−0.0027 (6)	−0.0022 (6)
C10	0.0189 (8)	0.0163 (7)	0.0184 (7)	−0.0054 (6)	0.0031 (6)	−0.0010 (6)
C11	0.0221 (8)	0.0147 (7)	0.0158 (7)	−0.0039 (6)	0.0011 (6)	0.0014 (6)
C12	0.0211 (8)	0.0160 (7)	0.0176 (8)	−0.0022 (6)	0.0007 (6)	−0.0006 (6)
C13	0.0191 (8)	0.0154 (7)	0.0154 (7)	−0.0026 (6)	−0.0014 (6)	−0.0001 (6)
C14	0.0218 (8)	0.0171 (7)	0.0154 (7)	−0.0030 (6)	−0.0023 (6)	−0.0033 (6)
C15	0.0191 (8)	0.0174 (8)	0.0202 (8)	−0.0053 (6)	−0.0039 (6)	−0.0010 (6)
C16	0.0255 (8)	0.0180 (8)	0.0260 (9)	−0.0049 (6)	−0.0044 (7)	−0.0035 (7)
C17	0.0298 (9)	0.0154 (8)	0.0321 (9)	−0.0072 (7)	−0.0064 (7)	0.0036 (7)
C18	0.0321 (9)	0.0231 (8)	0.0224 (8)	−0.0109 (7)	−0.0041 (7)	0.0071 (7)
C19	0.0259 (8)	0.0237 (8)	0.0188 (8)	−0.0094 (7)	−0.0009 (6)	0.0002 (6)
C20	0.0201 (8)	0.0169 (8)	0.0182 (8)	−0.0067 (6)	−0.0029 (6)	−0.0008 (6)
C21	0.0168 (7)	0.0182 (8)	0.0162 (7)	−0.0049 (6)	0.0007 (6)	−0.0016 (6)
C22	0.0250 (9)	0.0254 (9)	0.0292 (9)	0.0006 (7)	0.0008 (7)	0.0042 (7)
C23	0.0228 (8)	0.0185 (8)	0.0197 (8)	−0.0003 (6)	−0.0011 (6)	0.0015 (6)
C24	0.0205 (8)	0.0136 (7)	0.0216 (8)	0.0006 (6)	−0.0017 (6)	0.0033 (6)
C25	0.0234 (9)	0.0254 (9)	0.0283 (9)	−0.0049 (7)	−0.0070 (7)	0.0031 (7)
C26	0.0214 (9)	0.0253 (9)	0.0331 (10)	−0.0051 (7)	0.0010 (7)	0.0053 (7)
C27	0.0308 (9)	0.0203 (8)	0.0236 (9)	0.0016 (7)	0.0034 (7)	0.0048 (7)
C28	0.0335 (10)	0.0222 (8)	0.0241 (9)	0.0000 (7)	−0.0051 (7)	−0.0025 (7)
C29	0.0253 (9)	0.0156 (7)	0.0271 (9)	−0.0045 (6)	−0.0029 (7)	−0.0002 (6)
C30	0.0240 (9)	0.0318 (10)	0.0275 (9)	−0.0061 (7)	−0.0032 (7)	0.0012 (7)

### Geometric parameters (Å, °)

**Table:**

O1—C10	1.2158 (19)	C13—C21	1.539 (2)
O2—C14	1.4085 (19)	C13—C14	1.569 (2)
O2—H12O	0.82 (3)	C14—C15	1.511 (2)
O3—C21	1.2134 (19)	C15—C16	1.386 (2)
N1—C12	1.4682 (19)	C15—C20	1.397 (2)
N1—C8	1.470 (2)	C16—C17	1.391 (2)
N1—C14	1.485 (2)	C16—H16A	0.948 (19)
N2—C22	1.465 (2)	C17—C18	1.398 (3)
N2—C13	1.469 (2)	C17—H17A	0.96 (2)
N2—C30	1.478 (2)	C18—C19	1.385 (2)
C1—C2	1.383 (2)	C18—H18A	0.96 (2)
C1—C6	1.398 (2)	C19—C20	1.392 (2)
C1—H1A	1.01 (2)	C19—H19A	0.97 (2)
C2—C3	1.388 (3)	C20—C21	1.479 (2)
C2—H2A	1.03 (2)	C22—C23	1.528 (2)
C3—C4	1.388 (3)	C22—H22A	1.02 (2)
C3—H3A	0.97 (2)	C22—H22B	1.02 (2)
C4—C5	1.393 (2)	C23—C24	1.518 (2)
C4—H4A	0.93 (2)	C23—H23A	0.98 (2)
C5—C6	1.394 (2)	C24—C25	1.392 (2)
C5—H5A	1.01 (2)	C24—C29	1.392 (2)
C6—C7	1.471 (2)	C25—C26	1.391 (3)
C7—C9	1.344 (2)	C25—H25A	0.96 (2)
C7—H7A	1.05 (2)	C26—C27	1.380 (3)
C8—C9	1.528 (2)	C26—H26A	0.98 (2)
C8—H8A	0.96 (2)	C27—C28	1.386 (3)
C8—H8B	1.01 (2)	C27—H27A	1.00 (2)
C9—C10	1.497 (2)	C28—C29	1.388 (2)
C10—C11	1.523 (2)	C28—H28A	0.98 (2)
C11—C23	1.551 (2)	C29—H29A	0.96 (2)
C11—C12	1.557 (2)	C30—H30A	0.98 (2)
C11—C13	1.557 (2)	C30—H30B	1.03 (2)
C12—H12A	0.989 (19)	C30—H30C	1.06 (2)
C12—H12B	0.975 (19)		
			
C14—O2—H12O	105.0 (18)	O2—C14—C13	111.61 (13)
C12—N1—C8	109.09 (12)	N1—C14—C13	105.53 (12)
C12—N1—C14	101.76 (12)	C15—C14—C13	104.90 (12)
C8—N1—C14	115.23 (12)	C16—C15—C20	120.51 (14)
C22—N2—C13	105.68 (13)	C16—C15—C14	128.27 (15)
C22—N2—C30	111.93 (14)	C20—C15—C14	111.21 (13)
C13—N2—C30	115.98 (13)	C15—C16—C17	118.07 (16)
C2—C1—C6	120.81 (16)	C15—C16—H16A	118.9 (11)
C2—C1—H1A	120.0 (11)	C17—C16—H16A	123.0 (11)
C6—C1—H1A	119.2 (11)	C16—C17—C18	121.26 (15)
C1—C2—C3	120.10 (16)	C16—C17—H17A	118.4 (12)
C1—C2—H2A	119.9 (12)	C18—C17—H17A	120.4 (12)
C3—C2—H2A	120.0 (12)	C19—C18—C17	120.75 (15)
C2—C3—C4	119.93 (16)	C19—C18—H18A	120.1 (13)
C2—C3—H3A	120.5 (13)	C17—C18—H18A	119.2 (13)
C4—C3—H3A	119.6 (13)	C18—C19—C20	117.80 (16)
C3—C4—C5	119.85 (16)	C18—C19—H19A	122.1 (12)
C3—C4—H4A	120.6 (13)	C20—C19—H19A	120.1 (12)
C5—C4—H4A	119.6 (13)	C19—C20—C15	121.55 (15)
C4—C5—C6	120.66 (16)	C19—C20—C21	127.92 (15)
C4—C5—H5A	119.7 (12)	C15—C20—C21	110.52 (13)
C6—C5—H5A	119.6 (12)	O3—C21—C20	127.33 (14)
C5—C6—C1	118.62 (15)	O3—C21—C13	125.07 (14)
C5—C6—C7	122.39 (15)	C20—C21—C13	107.57 (12)
C1—C6—C7	118.99 (15)	N2—C22—C23	104.88 (14)
C9—C7—C6	126.85 (15)	N2—C22—H22A	106.9 (14)
C9—C7—H7A	117.9 (11)	C23—C22—H22A	116.5 (14)
C6—C7—H7A	115.1 (11)	N2—C22—H22B	109.0 (11)
N1—C8—C9	114.88 (13)	C23—C22—H22B	115.3 (11)
N1—C8—H8A	107.7 (12)	H22A—C22—H22B	104.0 (18)
C9—C8—H8A	111.2 (12)	C24—C23—C22	115.92 (14)
N1—C8—H8B	107.6 (11)	C24—C23—C11	112.96 (13)
C9—C8—H8B	108.8 (11)	C22—C23—C11	104.13 (13)
H8A—C8—H8B	106.1 (16)	C24—C23—H23A	108.3 (12)
C7—C9—C10	117.64 (14)	C22—C23—H23A	108.2 (12)
C7—C9—C8	124.61 (14)	C11—C23—H23A	106.8 (12)
C10—C9—C8	117.27 (13)	C25—C24—C29	118.25 (15)
O1—C10—C9	122.92 (15)	C25—C24—C23	119.22 (15)
O1—C10—C11	123.45 (14)	C29—C24—C23	122.50 (15)
C9—C10—C11	113.63 (13)	C26—C25—C24	121.00 (17)
C10—C11—C23	115.69 (13)	C26—C25—H25A	123.0 (13)
C10—C11—C12	105.08 (13)	C24—C25—H25A	115.8 (13)
C23—C11—C12	118.06 (13)	C27—C26—C25	120.17 (17)
C10—C11—C13	110.93 (12)	C27—C26—H26A	119.1 (12)
C23—C11—C13	105.36 (12)	C25—C26—H26A	120.7 (13)
C12—C11—C13	100.70 (12)	C26—C27—C28	119.42 (16)
N1—C12—C11	103.98 (12)	C26—C27—H27A	121.2 (13)
N1—C12—H12A	111.4 (11)	C28—C27—H27A	119.4 (13)
C11—C12—H12A	113.4 (11)	C27—C28—C29	120.43 (17)
N1—C12—H12B	110.0 (11)	C27—C28—H28A	121.6 (14)
C11—C12—H12B	108.6 (11)	C29—C28—H28A	117.9 (14)
H12A—C12—H12B	109.3 (15)	C28—C29—C24	120.71 (16)
N2—C13—C21	113.55 (13)	C28—C29—H29A	118.1 (13)
N2—C13—C11	103.47 (12)	C24—C29—H29A	121.2 (13)
C21—C13—C11	116.85 (12)	N2—C30—H30A	109.5 (12)
N2—C13—C14	113.34 (13)	N2—C30—H30B	113.5 (12)
C21—C13—C14	104.78 (12)	H30A—C30—H30B	103.0 (17)
C11—C13—C14	104.80 (12)	N2—C30—H30C	109.9 (11)
O2—C14—N1	108.21 (12)	H30A—C30—H30C	110.9 (17)
O2—C14—C15	111.55 (13)	H30B—C30—H30C	109.8 (16)
N1—C14—C15	114.89 (13)		
			
C6—C1—C2—C3	1.7 (3)	N2—C13—C14—N1	123.70 (13)
C1—C2—C3—C4	−0.1 (3)	C21—C13—C14—N1	−111.97 (13)
C2—C3—C4—C5	−1.1 (3)	C11—C13—C14—N1	11.59 (15)
C3—C4—C5—C6	0.6 (3)	N2—C13—C14—C15	−114.57 (14)
C4—C5—C6—C1	1.0 (2)	C21—C13—C14—C15	9.76 (15)
C4—C5—C6—C7	−179.61 (15)	C11—C13—C14—C15	133.32 (12)
C2—C1—C6—C5	−2.1 (2)	O2—C14—C15—C16	51.5 (2)
C2—C1—C6—C7	178.44 (15)	N1—C14—C15—C16	−72.2 (2)
C5—C6—C7—C9	46.8 (2)	C13—C14—C15—C16	172.45 (15)
C1—C6—C7—C9	−133.81 (18)	O2—C14—C15—C20	−128.10 (14)
C12—N1—C8—C9	48.56 (17)	N1—C14—C15—C20	108.28 (15)
C14—N1—C8—C9	−65.12 (17)	C13—C14—C15—C20	−7.11 (17)
C6—C7—C9—C10	173.33 (15)	C20—C15—C16—C17	−1.4 (2)
C6—C7—C9—C8	1.6 (3)	C14—C15—C16—C17	179.04 (16)
N1—C8—C9—C7	145.29 (15)	C15—C16—C17—C18	−0.7 (3)
N1—C8—C9—C10	−26.47 (19)	C16—C17—C18—C19	1.9 (3)
C7—C9—C10—O1	37.5 (2)	C17—C18—C19—C20	−0.9 (3)
C8—C9—C10—O1	−150.11 (15)	C18—C19—C20—C15	−1.3 (2)
C7—C9—C10—C11	−142.41 (14)	C18—C19—C20—C21	179.67 (16)
C8—C9—C10—C11	29.93 (18)	C16—C15—C20—C19	2.5 (2)
O1—C10—C11—C23	−4.6 (2)	C14—C15—C20—C19	−177.89 (14)
C9—C10—C11—C23	175.34 (12)	C16—C15—C20—C21	−178.31 (14)
O1—C10—C11—C12	127.48 (16)	C14—C15—C20—C21	1.28 (18)
C9—C10—C11—C12	−52.57 (15)	C19—C20—C21—O3	2.4 (3)
O1—C10—C11—C13	−124.53 (16)	C15—C20—C21—O3	−176.71 (15)
C9—C10—C11—C13	55.42 (17)	C19—C20—C21—C13	−175.60 (15)
C8—N1—C12—C11	−72.99 (15)	C15—C20—C21—C13	5.29 (17)
C14—N1—C12—C11	49.20 (14)	N2—C13—C21—O3	−63.2 (2)
C10—C11—C12—N1	74.48 (14)	C11—C13—C21—O3	57.2 (2)
C23—C11—C12—N1	−154.79 (13)	C14—C13—C21—O3	172.66 (15)
C13—C11—C12—N1	−40.83 (15)	N2—C13—C21—C20	114.90 (14)
C22—N2—C13—C21	89.21 (15)	C11—C13—C21—C20	−124.74 (14)
C30—N2—C13—C21	−35.42 (19)	C14—C13—C21—C20	−9.29 (16)
C22—N2—C13—C11	−38.45 (15)	C13—N2—C22—C23	41.02 (16)
C30—N2—C13—C11	−163.08 (14)	C30—N2—C22—C23	168.15 (14)
C22—N2—C13—C14	−151.36 (13)	N2—C22—C23—C24	98.82 (16)
C30—N2—C13—C14	84.00 (17)	N2—C22—C23—C11	−25.91 (17)
C10—C11—C13—N2	147.07 (13)	C10—C11—C23—C24	113.20 (15)
C23—C11—C13—N2	21.16 (15)	C12—C11—C23—C24	−12.5 (2)
C12—C11—C13—N2	−102.09 (13)	C13—C11—C23—C24	−123.89 (14)
C10—C11—C13—C21	21.49 (18)	C10—C11—C23—C22	−120.19 (15)
C23—C11—C13—C21	−104.42 (15)	C12—C11—C23—C22	114.10 (15)
C12—C11—C13—C21	132.33 (14)	C13—C11—C23—C22	2.72 (16)
C10—C11—C13—C14	−93.95 (14)	C22—C23—C24—C25	160.82 (15)
C23—C11—C13—C14	140.15 (12)	C11—C23—C24—C25	−79.13 (19)
C12—C11—C13—C14	16.89 (14)	C22—C23—C24—C29	−21.0 (2)
C12—N1—C14—O2	82.39 (14)	C11—C23—C24—C29	99.04 (18)
C8—N1—C14—O2	−159.73 (12)	C29—C24—C25—C26	−1.0 (2)
C12—N1—C14—C15	−152.24 (13)	C23—C24—C25—C26	177.28 (15)
C8—N1—C14—C15	−34.36 (18)	C24—C25—C26—C27	0.1 (3)
C12—N1—C14—C13	−37.20 (14)	C25—C26—C27—C28	1.0 (3)
C8—N1—C14—C13	80.67 (14)	C26—C27—C28—C29	−1.2 (3)
N2—C13—C14—O2	6.38 (17)	C27—C28—C29—C24	0.3 (3)
C21—C13—C14—O2	130.71 (13)	C25—C24—C29—C28	0.8 (2)
C11—C13—C14—O2	−105.73 (14)	C23—C24—C29—C28	−177.41 (15)

### Hydrogen-bond geometry (Å, °)

**Table:**

*D*—H···*A*	*D*—H	H···*A*	*D*···*A*	*D*—H···*A*
O2—H12O···N2	0.82 (3)	2.10 (3)	2.6741 (19)	127 (2)
C17—H17A···O1^i^	0.965 (19)	2.56 (2)	3.278 (2)	130.8 (15)
C26—H26A···O1^ii^	0.98 (3)	2.60 (2)	3.535 (2)	161.6 (18)
C29—H29A···O2^iii^	0.96 (3)	2.43 (2)	3.363 (2)	165.7 (18)

Symmetry codes: (i) *x*, *y*−1, *z*; (ii) −*x*+2, −*y*+2, −*z*; (iii) −*x*+1, −*y*+1, −*z*.
